# Role of Sr doping and external strain on relieving bottleneck of oxygen diffusion in La_2−*x*_Sr_*x*_CuO_4−δ_

**DOI:** 10.1038/s41598-022-17376-9

**Published:** 2022-08-04

**Authors:** Sohee Park, Young-Kyun Kwon, Mina Yoon, Changwon Park

**Affiliations:** 1 Department of Information Display, Kyung Hee University, Seoul, 02447 Korea; 2 Department of Physics and Research Institute for Basic Sciences, Kyung Hee University, Seoul, 02447 Korea; 3 Materials Science and Technology Division, Oak Ridge National Laboratory, Oak Ridge, TN 37831 USA; 4 School of Computational Sciences, Korea Institute for Advanced Study, Seoul, 130-722 Korea

**Keywords:** Fuel cells, Materials science, Theory and computation, Electronic structure

## Abstract

In many complex oxides, the oxygen vacancy formation is a promising route to modify the material properties such as a superconductivity and an oxygen diffusivity. Cation substitutions and external strain have been utilized to control the concentration and diffusion of oxygen vacancies, but the mechanisms behind the controls are not fully understood. Using first-principles calculations, we find how Sr doping and external strain greatly enhances the diffusivity of oxygen vacancies in La_2−*x*_Sr_*x*_CuO_4−δ_ (LSCO) in the atomic level. In hole-doped case (2*x* > δ), the formation energy of an apical vacancy in the LaO layer is larger than its equatorial counterpart by 0.2 eV that the bottleneck of diffusion process is for oxygen vacancies to escape equatorial sites. Such an energy difference can be reduced and even reversed by either small strain (< 1.5%) or short-range attraction between Sr and oxygen vacancy, and in turn, the oxygen diffusivity is greatly enhanced. For fully compensated hole case (2*x* ≦ δ), the formation energy of an apical vacancy becomes too high that most oxygen vacancies cannot move but would be trapped at equatorial sites. From our electronic structure analysis, we found that the contrasting change in the formation energy by Sr doping and external strain is originated from the different localization natures of electron carrier from both types of oxygen vacancies.

## Introduction

Oxygen vacancies in complex oxides drastically changes the electronic properties of them. One of the most studied examples is the superconducting critical temperature of La_2_CuO_4_^1–19^, for which both electron and hole doping have been achieved in conjunction with cation substitutions such as Ce^1^, Ca^2–6^, Sr^7–12^ and Ba^13–19^. Meanwhile, the oxygen stoichiometry in La_2_CuO_4_ can be reversibly controlled without degradation of the crystallinity^20^. This property not only makes the control of oxygen stoichiometry to be a promising route to control the carrier concentration^7,12,20–25^, but also makes complex oxides to be candidates for an electrolyte in solid oxide fuel cell^21,26–28^.

Cation substitutions and external strain has been utilized to control the electronic and diffusion properties of oxygen vacancies, but the mechanisms behind the controls are not fully understood. A part of the reason is that though the effects of oxygen vacancy strongly depend on crystallographic location (apical or equatorial) in its bulk structures, the measurements are performed in indirect ways such as Raman scattering^25^, lattice expansion^20^, and oxygen tracer diffusion^29^. In this work, to directly determine the crystallographic locations of oxygen vacancies in La_2−*x*_Sr_*x*_CuO_4_ (LSCO) and the role of them in the electronic and diffusion properties under external strain and doping, we scrutinize the energetic and electronic properties of oxygen vacancies using first-principles calculations. We found that the formation energies (${E}_{\mathrm{V}}$) of apical (${aV}_{\mathrm{O}}$) and equatorial oxygen vacancies (${eV}_{\mathrm{O}}$) depend strongly on doping concentrations and strain, but in different manners. Implications on the oxygen diffusivity and how external strain can effectively remove the bottleneck of oxygen diffusion in bulk LSCO will be discussed. From the vacancy level analysis, the contrasting change in the formation energy by Sr doping and external strain are from the different localization natures of electron carrier from apical and equatorial vacancy. This also indicate that it is nearly impossible to achieve electron doping by introducing oxygen vacancies.

## Theoretical approaches

We performed first-principles calculations using density functional theory (DFT)^30^ as implemented in Vienna ab initio simulation package (VASP)^31,32^. The spin-polarized generalized gradient approximation (SGGA) with Perdew–Burke–Ernzerhof (PBE) functional^33^ was used to treat the exchange and correlation functional. The electron wave functions were expanded by plane-wave basis with a kinetic energy cutoff of 400 eV. To introduce a low-temperature structure with alternatingly tilted octahedra (See "Atomic structure of La_2_CuO_4_" section and Table 1 for a detailed description of the structure), we used a $\left(2\sqrt{2}\times 2\sqrt{2}\right)R{45}^{^\circ }\times 2$ supercell containing 16 formula units and a 4 $\times$ 4 $\times$ 1 k-point mesh that uniformly samples the Brillouin zone (BZ) of the supercell. To obtain accurate densities of states, the BZ were sampled with a denser 8 $\times$ 8 $\times$ 4 k-point grid. All supercell structures were optimized until the force exerting on every atom became less than 0.02 eV/Å. The electron correlation effect associated with the Cu 3*d* orbitals was incorporated by the on-site Coulomb repulsion^34^ of *U*_eff_ = *U* – *J* = 4.0–8.0 eV^35–37^. We choose *U*_eff_ = 7.0 eV, which reproduces experimental Cu magnetic moment and band gap (See Table 1). Although ${E}_{\mathrm{V}}$ itself depends on *U*_eff_, the relative ${E}_{\mathrm{V}}$ between ${aV}_{\mathrm{O}}$ and $e{V}_{\mathrm{O}}$ is much less sensitive on the choice of *U*_eff_. For unstrained LSCO, we checked this using *U*_eff_ = 4.0 eV, which is from the reaction enthalpy of copper oxides^38^, confirming that our results on competitive formation of ${aV}_{\mathrm{O}}$ and $e{V}_{\mathrm{O}}$ does not depends on the choice of *U*_eff_ (see Supplementary Information Section 1). For the convenience of analysis, the lattice constant along the *c*-axis was fixed while applying the biaxial strain along the *a*- and *b*-axes. The lattice constant along the *c*-axis is affected by elastic moduli, Sr doping^39^ and oxygen vacancy concentration^20^, and the latter two effects are negligible in the vacancy formation energy. From our calculations, under the biaxial strain of ε (|ε|≤ 1.5%), *c*-axis is strained by -0.7 ε. Ignoring this elastic modulus effect slightly changes ${E}_{\mathrm{V}}$ in strained LSCO, but this amount is quite small (by definition, zero for unstrained LSCO) that our results do not be affected by the fixed c-axis calculations. (See Supplementary Information Section 1).

**Table 1:** Optimized structural parameters of La_2_CuO_4_ in the LTT phase calculated under -2.43%, 0%, and 2.61% biaxial strains.

Strain	−2.43%	0%	2.61%
$\sqrt{2}a$ (Å)	5.288	5.420 (5.360^42^)	5.561
$c$ (Å)	13.103	13.103 (13.151^42^)	13.103
${d}_{\mathrm{Cu}-a\mathrm{O}}$ (Å)	2.410	2.442	2.483
${d}_{\mathrm{Cu}-e\mathrm{O}}$ (Å)	1.870	1.916	1.966
${d}_{\mathrm{Cu}-e{\mathrm{O}}^{*}}$ (Å)	1.876	1.928	1.982
$\alpha$ (°)	4.6	7.4 (5.5–8.5^43^*)	9.6
$\mu$ (${\mu }_{B})$	0.585	0.603 (0.50—0.63^44,45^)	0.615
${E}_{g}$ (eV)	1.91	1.82 (1.80—2.00^46,47^)	1.75

Available experimental values were also listed in parenthesis with their corresponding references. Note that experimental references did not specify in what phases they measured their values. *Means the calculated value using various functionals.

## Results and discussion

### Atomic structure of La_2_CuO_4_

**Figure 1:**
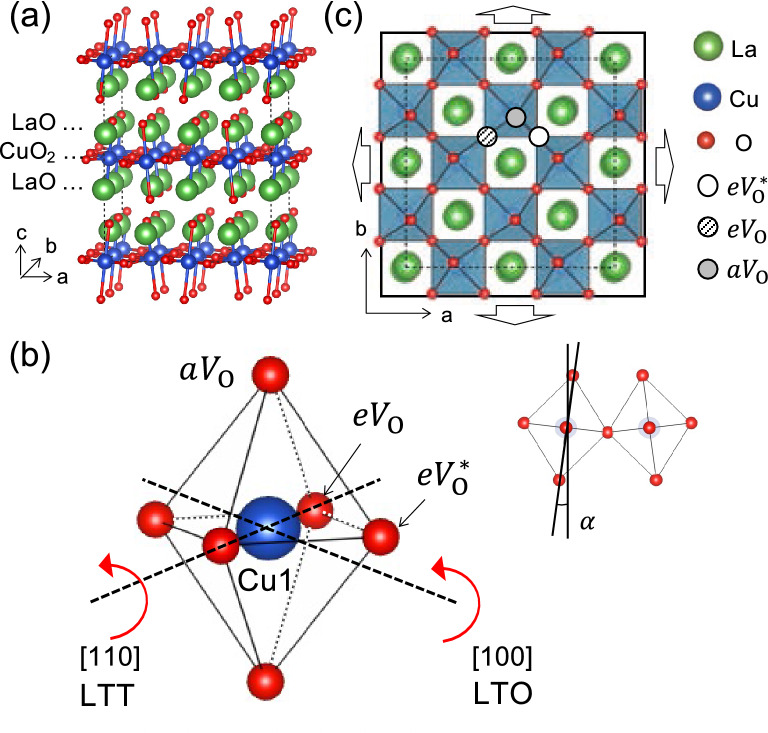
(**a**) La_2_CuO_4_ $\left(2\sqrt{2}\times 2\sqrt{2}\right)R{45}^{^\circ }\times 2$ structure composed of 16 formula units. The green, blue, and red balls represent lanthanum, copper, and oxygen atoms, respectively. (**b**) Two kinds of low-temperature phases are distinguished by the axis of octahedron tilt as indicated with red arrows. For LTT phase, CuO_6_ octahedron is tilted along [110] axis while for LTO phase, the tilting axis becomes [100]. Three inequivalent oxygens are indicated. α is tilting angle of the octahedron and its experimental and calculational values are listed in Table 1. (**c**) Top view of the LTT phase. White, hatched and gray circles are inequivalent locations of oxygen atoms termed as equatorial*(*eV*_O_^*^), equatorial (*eV*_O_) and apical site (*aV*_O_). Arrows indicate the applied uniform biaxial strain (the same scaling of lattice constant *a* and *b*).

La_2_CuO_4_ can be understood as a stacked structure of CuO_2_ layer (A) and LaO layers (B), with (AB)B(AB)B··· layers repeating as shown in Fig. 1a. As the temperature increases, La_2_CuO_4_ undergoes a successive phase transition from the low-temperature octahedral (LTO) phase to the low-temperature tetragonal (LTT) phase and then to the high-temperature tetragonal (HTT) phase. In the LTO and LTT phases, CuO_6_ octahedra are alternately tilted along the [100] and [110] axes (Fig. 1b), respectively, while they are presumably randomly tilted in the HTT phase. The total energy of the LTO phase is lower than the LTT phase by 4.0—4.4 meV per formula unit^40,43^, and the small energy difference can be easily reversed by cation doping^41^. Assuming the choice of a specific low-energy phase has little effect on the formation energy of oxygen vacancies, we adopt the LTT phase in our calculations. We constructed an optimized supercell structure of the LTT phase consisting of 16 formula units, as shown in Fig. 1a, with alternatingly-tilted octahedra (see Fig. 1c). In the LTT phase, there are three inequivalent oxygen sites: one apical site (${aV}_{\mathrm{O}}$) and two equatorial sites (${eV}_{\mathrm{O}}$, and $e{V}_{\mathrm{O}}^{*})$, with no and an out-of-plane displacement, respectively (see Fig. 1b). Table 1 summarizes our calculated lattice constants ($\sqrt{2}a$ and $c$), bond lengths ($d$) between Cu and each of the three oxygen vacancies, tilt angle ($\alpha$), Cu magnetic moment ($\mu$), and band gap (${E}_{g}$), which agree well with literature values^42–47^.

### Formation energy of oxygen vacancies as a function of Sr doping and biaxial strain

To explore the energetics of various oxygen vacancies in LSCO, we evaluated their formation energies ${E}_{\mathrm{V}}$ in a $\left(2\sqrt{2}\times 2\sqrt{2}\right)R{45}^{^\circ }\times 2$ supercell containing 16 formula units, defined as

$${E}_{\mathrm{V}}(n,{\mu }_{\mathrm{O}})={E}_{\mathrm{tot}}\left({\mathrm{La}}_{32-n}{\mathrm{Sr}}_{n}{\mathrm{Cu}}_{16}{\mathrm{O}}_{63}\right)-{E}_{\mathrm{tot}}\left({\mathrm{La}}_{32-n}{\mathrm{Sr}}_{n}{\mathrm{Cu}}_{16}{\mathrm{O}}_{64}\right)+{\mu }_{\mathrm{O}}$$

where ${E}_{\mathrm{tot}}$ is the total energy, $n$ is the number of Sr atoms in the supercell, and ${\mu }_{\mathrm{O}}$ is the oxygen chemical potential. We set ${{\mu }_{\mathrm{O}}\equiv \frac{1}{2}E}_{{\mathrm{O}}_{2}}=$ −4.392 eV where ${E}_{{\mathrm{O}}_{2}}$ is the formation energy of an oxygen molecule calculated with SGGA. We first checked the dependence of ${E}_{\mathrm{tot}}\left({\mathrm{La}}_{32-n}{\mathrm{Sr}}_{n}{\mathrm{Cu}}_{16}{\mathrm{O}}_{64}\right)$ on the Sr configurations. Specifically, we found for $n=$ 2, ${E}_{\mathrm{tot}}\left({\mathrm{La}}_{32-n}{\mathrm{Sr}}_{n}{\mathrm{Cu}}_{16}{\mathrm{O}}_{64}\right)$ becomes lower as two Sr atoms are getting closer, but the maximum energy difference is 28 meV, which is quite weak for Sr atoms to form any type of ordering during the high-temperature process of synthesis. On the other hands, between Sr atom and oxygen vacancy, much larger attractive interaction exists. At zero strain with $n=$ 2, ${E}_{V}$ of ${aV}_{\mathrm{O}}$ is 1.98 eV when Sr is far away for ${aV}_{\mathrm{O}}$ (6.09 Å), and it becomes lower to 1.76 eV when Sr is at the nearest location with ${aV}_{\mathrm{O}}$ (2.59 Å). For ${eV}_{\mathrm{O}}$, the attraction is smaller and short-ranged that ${E}_{V}$ changes from 1.77 eV to 1.71 eV only when Sr atom is the nearest neighbor of ${eV}_{\mathrm{O}}$ (2.70 Å). More details can be found in Supplementary Information Section 3. The strength of Sr-vacancy interaction is strong enough to affect the competitive formation of ${eV}_{\mathrm{O}}$ and ${aV}_{\mathrm{O}}$ at hole-doped sample as we will see soon.

**Figure 2:**
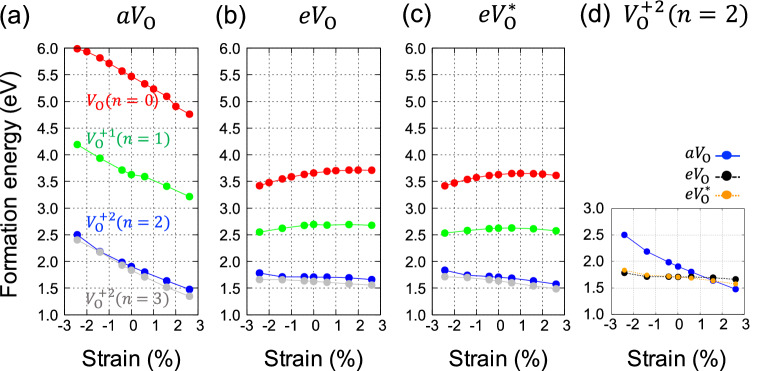
Oxygen vacancy formation energy (${E}_{V}$) in La_32−n_Sr_n_Cu_16_O_63_ under the strain from -2.43% to 2.61%. Red, green, blue, and gray colors correspond to *n* = 0, 1, 2, and, 3, respectively. Note that maximum oxidation number of oxygen vacancy is + 2. ${E}_{V}$’s of (**a**) apical and (**b**), (**c**) two inequivalent equatorial sites indicated in Fig. 1(c) are plotted. (**d**) ${E}_{V}$’s of + 2 charged vacancies (*n* = 2) are plotted for the comparison.

Figure 2 summarizes the ${E}_{V}$’s of three types of oxygen vacancies (${aV}_{\mathrm{O}}$, ${eV}_{\mathrm{O}}$, and $e{V}_{\mathrm{O}}^{*}$) for $n=$ 0, 1, 2, and 3 cases as a function of strain. For all ${E}_{V}$’s, vacancies are placed as far as possible from Sr atoms in our supercell (larger than 8 Å). The almost indistinguishable ${E}_{V}$’s of ${eV}_{\mathrm{O}}$ and $e{V}_{\mathrm{O}}^{*}$ (Fig. 2b,c) indicate the effect of octahedral tilt is negligible and we will focus only on $e{V}_{\mathrm{O}}^{*}$ from now on. Sr atoms provide $n$ holes per supercell and they recombine with the oxygen vacancy making it a charged vacancy. As $n$ increases, regardless of oxygen sites and strain, ${E}_{V}$ greatly decreases until $n=$ 2, while further Sr doping beyond $n=$ 2 scarcely change ${E}_{V}$, indicating the stability of + 2 charged vacancy (${V}_{\mathrm{O}}^{+2}$). Interestingly, the energy difference between $e{V}_{\mathrm{O}}^{*}$ and ${aV}_{\mathrm{O}}$ in 0% strain case significantly reduces from 1.84 eV in the undoped sample to 0.20 eV in the sample with the + 2 charge states. The general trend that increasing Sr doping greatly reduces ${E}_{V}$ until $n=$ 2 can be understood as follows. Once an oxygen vacancy forms, formally two electrons are released and they are localized around the vacancy. They now lose the energy gain from the large electron affinity of oxygen that ${E}_{V}$ is strongly affected by where and how tightly these two electrons are localized. In this sense, a Sr dopant (substituting La) can be thought to provides a location with a large electron affinity for one electron. The reason why the reduction of ${E}_{V}$ on Sr doping is so different for ${aV}_{\mathrm{O}}$ and ${eV}_{\mathrm{O}}$ can be explained with the different localization natures of electrons, and the detailed analysis will be presented in "Charge distribution and structural distortion by oxygen vacancies" section.

Our results imply that most oxygen vacancies in the undoped sample are located at equatorial sites. In the view of diffusion process via the vacancy mechanism, the bottleneck for the bulk diffusion is the equatorial-to-apical sites hopping, which is the only channel for diffusion along the c-axis. This hopping is nearly impossible to occur at the operation temperature of practical solid oxide fuel cells (< 1000 °C) because the lower bound of activation barrier is the relative ${E}_{V}$ between ${aV}_{\mathrm{O}}$ and ${eV}_{\mathrm{O}}$ (> 1 eV). However, in the hole-doped sample, oxygen vacancy can hop between equatorial and apical sites due to the reduced formation energy difference, and for zero strain and $n=$ 2 case, calculated energy barrier between apical-to-equatorial site is 0.52 eV. The attractive interaction between Sr and ${aV}_{\mathrm{O}}$ reduces ${E}_{V}$ of ${aV}_{\mathrm{O}}$, and in turn, it further reduces the energy barrier up to ~ 0.1 eV when a Sr atom is at the nearest neighbor of ${aV}_{\mathrm{O}}$. Experimentally, the formation of ${aV}_{\mathrm{O}}$ in hole-doped samples was evidenced by confocal Raman microscopy^25^. Though the signal is interpreted as selective formation of apical vacancies, we think further investigations are called for to confirm it because from our calculation, the energy difference between both types of vacancies is not so large for the prevalence of a specific type.

The effect of strain on ${E}_{V}$ is quite different for $e{V}_{\mathrm{O}}^{*}$ and ${aV}_{\mathrm{O}}$. In the case of ${aV}_{\mathrm{O}}$, ${E}_{V}$ decreases linearly with a tensile strain at a similar rate for different Sr-doped samples. On the contrary,${E}_{V}$ of $e{V}_{\mathrm{O}}^{*}$ shows relatively weak dependence on the strain and even non-monotonic behavior for neutral and + 1 charged vacancies (${V}_{\mathrm{O}}^{+1}$). This difference is mainly from the distinctive bonding nature of apical and equatorial oxygen atom where the former is more ionic. We will investigate this further in "Charge distribution and structural distortion by oxygen vacancies" section by analyzing the structural distortions and accompanying internal stress from the oxygen vacancies. The small energy difference between + 2 charged ${aV}_{\mathrm{O}}$ and $e{V}_{\mathrm{O}}^{*}$ is further reduced when biaxial strain is applied and even reversed at about 1–1.5% of strain as shown in Fig. 2d, which is achievable for epitaxially grown samples. For La_1.85_Sr_0.15_CuO_4_, it was clearly demonstrated that the tensile strain greatly accelerates reversible redox reaction^20^. Because the amount of reduction in ${E}_{V}$ on the biaxial strain is not so drastic for explaining the experiments, the acceleration was attributed to the surface exchange kinetics^20^. But for the whole sample to be uniformly doped, the rate-determining step should be the bulk diffusion including equatorial-to-apical hopping process. Our calculation indicates that the major role of tensile strains is to reduce ${E}_{V}$ of ${aV}_{\mathrm{O}}$, which does in turn, make the hopping process easier.

### Charge distribution and structural distortion by oxygen vacancies

**Figure 3:**
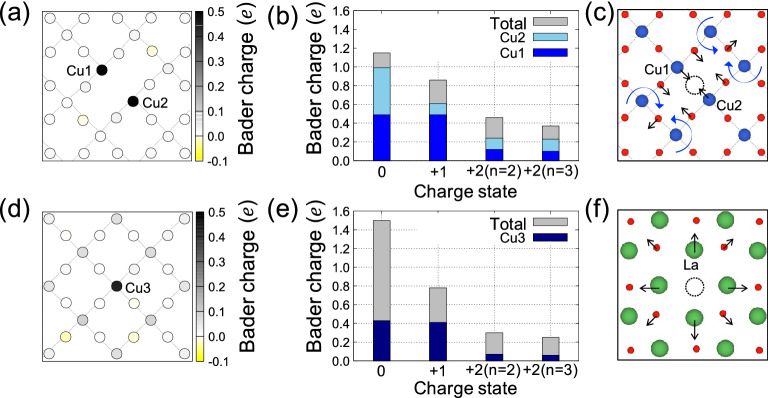
Atom-resolved Bader charge differences of (**a**) neutral *eV*_O_^*^ and (**d**) neutral *aV*_O_ relative to pristine La_2_CuO_4_. The scale bar indicates the amounts of charge accumulation (black) or depletion (yellow). For various charges states of (**b**) *eV*_O_^*^ and (e) *aV*_O_, each Cu’s contribution to Bader charge difference are shown as stacked histogram. Atomic relaxation around (**c**) *eV*_O_^*^ and (f) *aV*_O_. Each black arrow indicates the displacement of the corresponding atom with respect to its original position in the pristine structure. The curved blue arrows in (**c**) depict the rotation of the corresponding octahedra.

Once an oxygen vacancy forms, formally two electrons are released and they are weakly bound at (or transferred to) nearby Cu atoms having *d*^9^ configurations. This can be quantified using Bader charge analysis^48^ where total electron charge is divided and assigned to each atom according to Bader’s zero flux surface scheme. The assigned charge is called Bader charge and useful for tracing the charge transfer of a system. For $e{V}_{\mathrm{O}}^{*}$, 1.15 electrons are transferred from oxygen atoms to Cu atoms, and 86% of them uniformly occupy the vacant *d*-orbitals of the two nearest Cu atoms, as shown in Fig. 3a. This results in a complete suppression of the magnetic moments of those two Cu atoms. Figure 3b shows charge allocations to the two Cu atoms for different charge states. For + 1 charge state, due to the strong on-site Coulomb repulsion of the Cu *d*-orbitals, only one of two copper atoms loses its electron, breaking the symmetric charge distribution. For + 2 charge states, two Cu atoms return to original *d*^9^ configurations, recovering their magnetic moment (0.579 ${\mu }_{B}$) almost to the pristine bulk value (0.603 ${\mu }_{B}$). The structural distortion caused by $e{V}_{\mathrm{O}}^{*}$ is mainly from a weak attraction between the two Cu atoms (Cu1 and Cu2). As shown in Fig. 3c, the weak attraction causes two Cu atoms to approach each other and simultaneously rotate the neighboring octahedra, indicated by the blue arrows. The interatomic distance between the two Cu atoms is smallest for neutral $e{V}_{\mathrm{O}}^{*}$ (3.511 Å) and gradually recovers to the pristine bulk value (3.832 Å) as being positively charged (specifically, 3.757 Å for $e{V}_{\mathrm{O}}^{+2}$).

For ${aV}_{\mathrm{O}}$, one of the two released electrons from the oxygen vacancy is captured by Cu3 atom at the bottom of the vacancy and the remaining one is transferred to the lower CuO_2_ plane and spatially extended as shown in Fig. 3d. This is somewhat contrasted to F-center in an ionic crystal where the released electrons from an anionic vacancy are well localized at the vacancy. Within Bader charge analysis (Fig. 3e), 1.51 electrons are transferred from the oxygen atom and only a third of them occupy the hole state of the bottom Cu3 atom in no charge state. For + 1 charge state, the extended states first recombine with the hole, whereas for + 2 charge states, Cu3 recovers its magnetic moment, 0.580 ${\mu }_{B}$. This charge redistribution causes copper layers and octahedrons to be gradually rearranged and makes the structure unstable. The absence of the negative oxygen ion causes the four positive lanthanum ions to repel each other by 0.334 Å, as shown in Fig. 3f. Similar with ${eV}_{\mathrm{O}}$, vacancy charging mitigates the structural distortions around oxygen vacancies.

As shown in Fig. 2a, ${E}_{\mathrm{V}}$ of ${aV}_{\mathrm{O}}$ decrease linearly with tensile strain at a similar rate regardless of their charge states. In most ionic crystals and perovskite materials such as BaTiO_3_, SrTiO_3_, SrZrO_3_, and PbZrO_3_, cations around anionic vacancy move away from each other^49^, indicating the strong Coulomb repulsions between the cations. This induces strong local compressive stress in the crystal and if the crystal is allowed to relax, the equilibrium lattice constants along the *a*- and *b*-axes increase by 0.6% for *n* = 0 case of our supercell geometry. The dependence on charge states is minute since the electron carriers from ${aV}_{\mathrm{O}}$ are away from the apical sites that they play little role in reducing the Coloumb repulsion energy between the La atoms. Becase the tensile strain can mitigate the local compressive stress, this explains the decrease in ${E}_{\mathrm{V}}$ of ${aV}_{\mathrm{O}}$ on tensile strain at a similar rate regardless of their charge states.

On the other hand, ${E}_{\mathrm{V}}$'s of $e{V}_{\mathrm{O}}^{*}$ (or ${eV}_{\mathrm{O}})$ show a relatively weak, non-monotonic dependence on both strain and charge states, as shown in Fig. 2c. In CuO_2_ plane, the attraction between two Cu atoms around $e{V}_{\mathrm{O}}^{*}$ induces local tensile stress while in the neighboring two LaO planes, small but finite compressive stress is present, which we can infer from the displacement of La atoms. The non-monotonic behavior comes from the competition of the two opposite stresses. As $e{V}_{\mathrm{O}}^{*}$ is positively charging, the attraction becomes weaker and for + 2 charge states, compressive stress in LaO planes prevails and this explains weak linear decrease of ${E}_{\mathrm{V}}$ on tensile biaxial strain.

### Electronic structures of oxygen vacancy states

**Figure 4:**
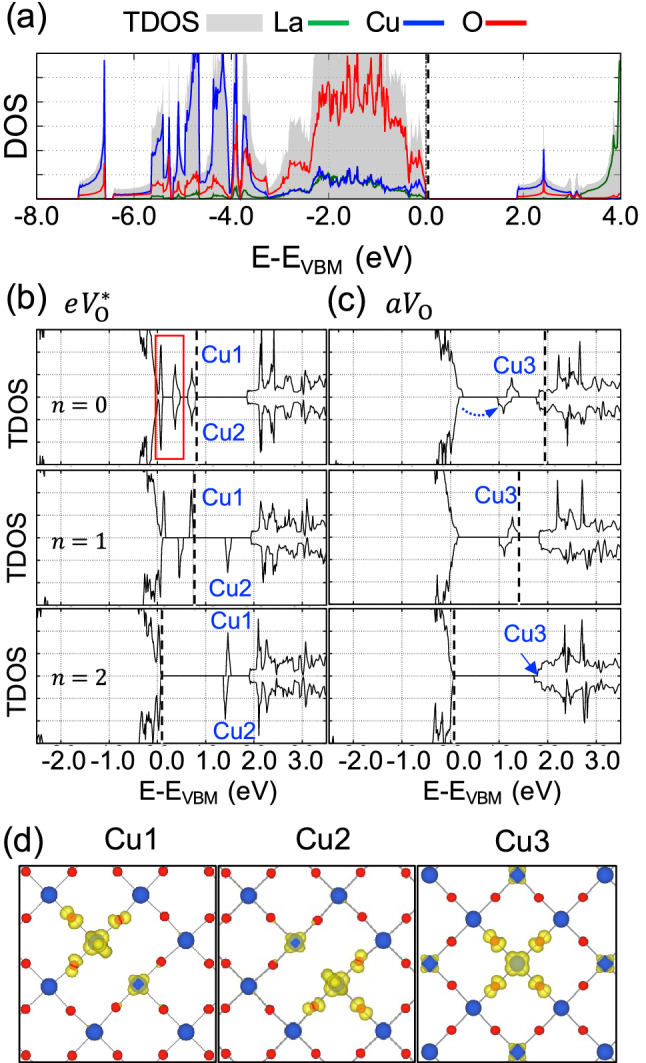
(**a**) Total density of states (TDOS) (grey filled curve) and projected density of state (PDOS) (color lines) on La, Cu, and O of La_2_CuO_4_. Spin-resolved TDOS of La_2_CuO_4_ with (**b**) *eV*_O_^*^ and (**c**) *aV*_O_ for various charge states. The scale of TDOS is the same for all six plots of (**b**) and (**c**). Oxygen vacancy states are indicated Cu1, Cu2, and Cu3. The dashed vertical line in each figure indicates the Fermi energy. States in the red rectangle of (**b**) and indicated with dotted arrow in (**c**) are from the occupied *d*^9^ states of Cu. (**d**) Spatial distributions of charge densities of three states marked in (**b**) and (**c**).

Figure 4a shows the total density of states (TDOS) and projected density of states (PDOS) of La_2_CuO_4_. From orbital-projected DOS analysis (not shown here), it was found that the fully occupied oxygen 2*p* orbitals are hybridized with the Cu 3*d* orbitals located near the top of valence band, while the ${d}_{{x}^{2}- {y}^{2}}$ hole states of Cu form the bottom of the conduction band. In Fig. 4b, $e{V}_{\mathrm{O}}^{*}$ induces two vacancy states (Cu1 and Cu2 states) at 0.8 eV above the valence band maximum. The energy level of the *d*^9^ states of the two nearest Cu atoms from the vacancy is also changed due to the breaking of the Cu–O bond, which are manifested as the four in-gap states indicated by a red rectangle in Fig. 4b. The distribution of two vacancy states are plotted in Fig. 4d. It clearly shows that the additional electrons are localized in the Cu atoms. If $e{V}_{\mathrm{O}}^{*}$ is singly charged, one of the Cu states becomes unoccupied and the energy degeneracy of *d*^9^ states are slightly lifted as shown in Fig. 4b. As explained in "Formation energy of oxygen vacancies as a function of Sr doping and biaxial strain", if the sample is hole-doped and oxygen vacancy is less than half of the hole concentration, most electrons in the vacancy states recombine with two holes leaving two unoccupied in-gap states, as in *n* = 2 case.

In the case of ${aV}_{\mathrm{O}}$, one of two released electron forms a vacancy state associated with Cu3 at 1.3 eV above the valence band maximum while the other electron does a shallow level which is very close to the conduction band minimum as shown in Fig. 4c. Though ${aV}_{\mathrm{O}}$ seems to play an electron donor, ${E}_{\mathrm{V}}$ of it is very high compared to that of $e{V}_{\mathrm{O}}^{*}$ indicating that ${aV}_{\mathrm{O}}$ is hard to form, or fast diffuses into the equatorial sites. In other words, oxygen vacancy in LSCO can compensate the holes in the sample but electron doping by overcompensation ($2\delta >x$) may not occur. Charge distribution of the vacancy state (Cu3) is shown in Fig. 4d. Other in-gap states in down spin channel come from one of the Cu3 *d*^9^ states due to Cu–O bond breaking (blue dotted arrow). If ${aV}_{\mathrm{O}}$ is singly charged, the electron in the shallow level first recombines with the hole. In the + 2 charge state, unlike $e{V}_{\mathrm{O}}^{*}$, hole recombination makes Cu3 state to merge into conduction band, leaving no in-gap states.

## Conclusion

We have calculated ${E}_{\mathrm{V}}$ of $e{V}_{\mathrm{O}}^{*}$ and ${aV}_{\mathrm{O}}$ in LSCO considering their charge states and strain dependence. In hole-doped cases, most oxygen vacancies become + 2 charge states and compensate the hole density regardless of crystallographic location. ${E}_{\mathrm{V}}$ of $e{V}_{\mathrm{O}}^{*}$ is lower than that of ${aV}_{\mathrm{O}}$ by 0.2 eV, but the small energy difference can be easily overcome by 1.5% of tensile strain or Sr-${aV}_{\mathrm{O}}$ attractions, enhancing the oxygen diffusivity in Sr-overdoped samples. Even though the number of vacancies is sufficiently large to fully compensate the holes, the sample is hard to be electron-doped because the electrons from the equatorial vacancies are strongly bound around the vacancies. Neutral ${aV}_{\mathrm{O}}$ can be an electron donor due to shallow vacancy level, but ${E}_{\mathrm{V}}$ of it is too high to form sufficient oxygen vacancy at apical sites. The possible coexistence of both types of vacancies in LSCO, especially in the epitaxially strained film, calls for careful interpretation of the vacancy-induced effect in the electronic structure of LSCO, such as the critical temperature of superconductors.

## Supplementary Information

Supplementary Information.

## Data Availability

The datasets used or analysed during the current study are available from the corresponding author on reasonable request.
