# Supplementary material for: Role of Sr doping and external strain on relieving bottleneck of oxygen diffusion in La2−xSrxCuO4−δ
Source: Sci Rep. 2022 Aug 4;12:13378. doi: 10.1038/s41598-022-17376-9 (PMC9352678; doi:10.1038/s41598-022-17376-9)
Supplement: Supplementary file 1 — Supplementary Information. [file 41598_2022_17376_MOESM1_ESM.docx]

**Supplementary Information on**

**“Role of Sr doping and external strain on relieving bottleneck of oxygen diffusion in La_2-_*_x_*Sr*_x_*CuO_4-𝛿_”**

Sohee Park^1^, Young-Kyun Kwon^1,2^, Mina Yoon^3^, Changwon Park^4^

*^1^ Department of Information Display, Kyung Hee University, Seoul 02447, Korea*

*^2^ Department of Physics and Research Institute for Basic Sciences, Kyung Hee University,
Seoul 02447, Korea*

*^3^ Materials Science and Technology Division, Oak Ridge National Laboratory,
Oak Ridge, TN 37831, United States,*

*^4^ School of Computational Sciences, Korea Institute for Advanced Study, Seoul 130-722, Korea*

**1. Effect of on-site Coulomb repulsion on the vacancy formation energy**

The electron correlation effect associated with the Cu 3d orbitals was incorporated by the on-site Coulomb repulsion [34] *U*_eff_ = *U*-*J*. The values of *U*_eff_ is determined to reproduce experimental observables such as Cu magnetic moment and band gap (*U*_eff_ = 7.0 eV), or the reaction enthalpy of copper oxides [38] (*U*_eff_ = 4.0 eV). In Fig. S1, we showed the choice of *U*_eff_ affects the formation energy of oxygen vacancies, but the energy difference between apical and equatorial vacancies is much less sensitive than the formation energy itself.

Figure S1. Formation energy of apical (${aV}_{O}$) and equatorial vacancies ($eV_{O}^{*})$ for *U*_eff_ = 7.0 and 4.0 eV. *n* is the number of Sr atoms in a La_2-_*_x_*Sr*_x_*CuO_4-𝛿_ supercell with 16 formular units, and formation energies are calculated when one oxygen vacancy is present in the supercell. The energy differences between ${aV}_{O}$ and $eV_{O}^{*}$ (open and filled triangles) are plotted for a comparison.

**2. Effect of C-axis lattice constant optimization on the vacancy formation energy**

In Fig. S2(a), we show the change in the *c*-axis lattice constant by Sr doping and oxygen vacancy formation. In the absence of Sr doping, oxygen vacancies slightly shrink the *c*-axis (red dots) while in the presence of Sr doping, they expand the c-axis (blue dots). In any cases, the change is at most 0.4%. Corresponding changes in formation energy is summarized in Table S1. From our calculations, on applying biaxial strain of ε, lattice constant along *c*-axis changes by -0.7ε (not shown here). Considering this effect slightly modifies the formation energies of vacancies at finite strain (Fig. S2(b)). Because tensile strain reduces the formation energy of ${aV}_{O}$ (black filled and blue open circle) and increases the formation energy of $eV_{O}^{*}$ (gray filled and red open circle), the required strain for ${aV}_{O}$ in hole-doped LSCO to have the same formation energy with $eV_{O}^{*}$ in hole-doped LSCO becomes slightly smaller if we consider the *c*-axis optimization effect.

Figure S2. (a) Effects of Sr doping and oxygen vacancy formation on the change of *c*-axis lattice constant. (b) Effect of biaxial strain on the formation energies of oxygen vacancies in LCO.

|  | La_32_Cu_16_O_63_ | |  | La_30_Sr_2_Cu_16_O_63_ | |
| --- | --- | --- | --- | --- | --- |
|  | $eV_{O}^{*}$ | $aV_{O}$ |  | $eV_{O}^{*}$ | $aV_{O}$ |
| Fixed  *c*-axis | 3.630 | 5.470 |  | 1.705 | 1.905 |
| Optimized  *c*-axis | 3.628 | 5.469 |  | 1.717 | 1.918 |

(unit: eV)

Table S1. Effect of c-axis optimization on the oxygen vacancy formation energy for neutral (La_32_Cu_16_O_63_) and +2 charge states (La_30_Sr_2_Cu_16_O_63_).

**3. Effect of Sr environment on the formation energy of oxygen vacancy**

The formation energy $E_{V}$ of oxygen vacancy depends not only on the crystallographic location but also on the Sr environment, and in general, $E_{V}$ becomes lower when Sr atoms is nearby. Figure S3 shows various oxygen vacancies locations in $\left( 2\sqrt{2}\times2\sqrt{2} \right)R{45}^{^{\circ}}\times2$ supercell when two Sr atoms substitute La atoms at zero strain. For apical vacancies, $E_{V}$ gradually increase as their distance increases and is saturated at location 4 (6.09 Å) to 1.98 eV. For equatorial vacancies, the attraction is smaller and short-ranged that $E_{V}$ changes from 1.77 eV to 1.71 eV only when Sr atom is the nearest neighbor of ${eV}_{O}$ (location 7).


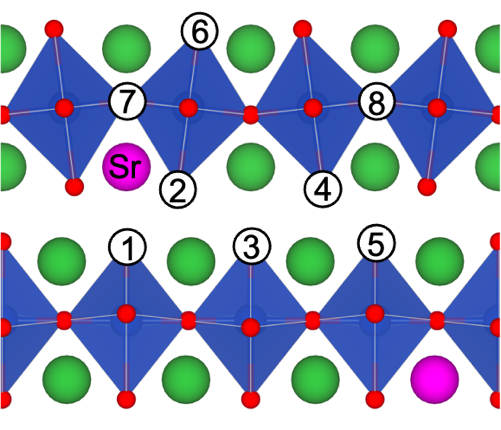


| Location | Distance from Sr ($Å$) | Formation energy (eV) |
| --- | --- | --- |
| 1 | 2.59 | 1.76 |
| 2 | 2.69 | 1.82 |
| 3 | 4.19 | 1.91 |
| 4 | 6.09 | 1.98 |
| 5 | 7.66 | 1.98 |
| 6 | 4.96 | 1.93 |
| 7 | 2.70 | 1.71 |
| 8 | 6.41 | 1.77 |

Figure S3. Formation energies of oxygen vacations when Sr atoms are nearby. Location 1-6 (7-8) correspond apical (equatorial) oxygen vacancies. Two Sr atoms (magenta) substituting La atoms (green) in $\left( 2\sqrt{2}\times2\sqrt{2} \right)R{45}^{^{\circ}}\times2$ supercell are shown.
